# PEGylation of zinc nanoparticles amplifies their ability to enhance olfactory responses to odorant

**DOI:** 10.1371/journal.pone.0189273

**Published:** 2017-12-20

**Authors:** Melissa Singletary, Samantha Hagerty, Shin Muramoto, Yasmine Daniels, William A. MacCrehan, Gheorghe Stan, June W. Lau, Oleg Pustovyy, Ludmila Globa, Edward E. Morrison, Iryna Sorokulova, Vitaly Vodyanoy

**Affiliations:** 1 Department of Anatomy, Physiology and Pharmacology, Auburn University College of Veterinary Medicine, Auburn, Alabama, United States of America; 2 Material Measurement Laboratory, National Institute of Standards and Technology, Gaithersburg, Maryland, United States of America; Institute of Materials Science, GERMANY

## Abstract

Olfactory responses are intensely enhanced with the addition of endogenous and engineered primarily-elemental small zinc nanoparticles (NPs). With aging, oxidation of these Zn nanoparticles eliminated the observed enhancement. The design of a polyethylene glycol coating to meet storage requirements of engineered zinc nanoparticles is evaluated to achieve maximal olfactory benefit. The zinc nanoparticles were covered with 1000 g/mol or 400 g/mol molecular weight polyethylene glycol (PEG). Non-PEGylated and PEGylated zinc nanoparticles were tested by electroolfactogram with isolated rat olfactory epithelium and odorant responses evoked by the mixture of eugenol, ethyl butyrate and (±) carvone after storage at 278 K (5 ^o^C), 303 K (30 ^o^C) and 323 K (50 ^o^C). The particles were analyzed by atomic force microscopy, transmission electron microscopy, X-ray photoelectron spectroscopy, and laser Doppler velocimetry. Our data indicate that stored ZnPEG400 nanoparticles maintain physiologically-consistent olfactory enhancement for over 300 days. These engineered Nanoparticles support future applications in olfactory research, sensitive detection, and medicine.

## Introduction

Metal nanoparticles have been isolated from the blood of several vertebrate species, leading to investigations regarding their role within the olfactory system [1]. Among the group of elemental metals, zinc demonstrated a significant enhancement of olfactory responses to odorant stimulation not seen with copper, gold, or silver [2]. Zinc nanoparticles were engineered using a high-voltage electrical discharge method. For electrophysiology, the olfactory epithelium (OE) was surgically removed from adult Sprague-Dawley rats, and whole cell patch-clamp and electroolfactogram (EOG) were evoked by a headspace vapor of a 1.6 mmole/L standard odorant solution of ethyl butyrate, eugenol, and ±carvone with or without zinc nanoparticles to be tested [2]. By these *ex vivo* experiments, enhancement was found to be dose-dependent, specific, and reversible. In conjunction with *ex vivo* analyses of sensory neurons in the rodent OE, we noninvasively analyzed *in vivo* cognitive effects on the brain regions associated with olfaction of non-anesthetized dogs using functional magnetic resonance imaging (fMRI). These studies indicated that engineered zinc nanoparticles added to odorant caused a significant rise of olfactory associated brain activity [3].

In a comparison of engineered zinc nanoparticles of various sizes and states of oxidation, the highest olfactory enhancement was noted for nanoparticles measuring approximately 1.2 nm in diameter and in the primarily-elemental state, while oxidation inhibited the enhancement [4]. However, the enhancement declined over time. Therefore, improvement of the nanoparticles was necessary. In this work, we tested the hypothesis that PEGylation of the zinc nanoparticles using 1000 g/mol or 400 g/mol molecular weight polyethylene glycol (PEG) would afford retention of the olfactory enhancement. The PEGylation is a chemical process involving the passivation of the surface of nanoparticles by PEG, a coiled polymer with multiple ethylene ether units that are soluble in water [5]. The PEGylation apparently decreases the rate of oxidation. It can provide an increased affinity to the target protein and reduced cytotoxicity of nanoparticles [5]. The PEGylation is broadly used for protection of nanoparticles against aggregation in the biological and pharmaceutical applications [6, 7]. Although there are many publications on the PEGylated zinc oxide nanoparticles [8, 9], this is the first study of PEGylated elemental zinc nanoparticles.

Small metal and metal composite nanoparticles have certain advantages compared to other particles due to their capability to specifically interact with proteins, peptides, and nucleic acids [10] and serve as important players in nanoparticle-based drugs, [11] as metal electrocatalysts, [12] in immune quantum dots [13], by crossing biological barriers [14], and many other applications in nanomedicine [15].

The protection of metal nanoparticles from rapid oxidation and improvement of their biophysical and biochemical properties for better interactions with receptors, compatibility of the particle-cell interface, and reduced toxicity, is important for their potential clinical outcome [10, 16, 17].

We characterized the PEGylated nanoparticles using scanning electron microscopy (SEM), transmission electron microscopy (TEM), atomic force microscopy (AFM), X-ray photoelectron spectroscopy (XPS), and laser Doppler velocimetry (LDV).

The overall goal of this work was to protect zinc nanoparticles from oxidation and preserve their physiological properties to enhance olfactory responses to odorants over the long storage and elevated temperatures.

The future outcome of this work could provide an improvement in the individual sense of smell in conditions such as Alzheimer’s and Parkinson’s, which demonstrate olfaction deficits [18]. In early Alzheimer’s olfactory deficits are a preclinical sign that worsens with the disease progression [19, 20]. Alzheimer’s disease affects an estimated 5.5 million Americans as of 2017 and is the 10th leading cause of death in the United States [18]. We hope that future treatments with zinc nanoparticles acting on the olfactory receptor level at minimal concentrations could reverse the loss of smell and improve emotional well-being and quality of life.

## Materials and methods

To characterize the properties of zinc nanoparticles under varying conditions, two sets of experimental settings were utilized: (1) Non-PEGylated and PEGylated zinc nanoparticles were stored at 283 K (5 ^o^C) and then employed, along with a standard odorant mixture in EOG olfactory experiments over a storage period of 317 days. (2) Non-PEGylated and PEGylated zinc nanoparticles were subjected to accelerated aging at temperatures of 303 K (30 ^o^C) and 323 K (50 ^o^C) for 2 days before the EOG experiments that followed directly after storage.

### Preparation of PEGylated Zn nanoparticles

Zinc nanoparticles of ~1.2 nm in size were prepared by a high-voltage electrical discharge method [21]. The of polyethylene glycol solutions of molecular weight 1000 g/mol or 400 g/mol (Sigma-Aldrich), PEG1000 or PEG400, respectively, were added to the nanoparticle suspension to make 1% w/v of PEGs. The suspensions were heated to 40 ^o^C, purged with N_2_ obtained from liquid nitrogen, and maintained at these conditions for 20 minutes. The suspension was sonicated at 19 W, 40 kHz for 20 min.

### TEM

TEM was carried out utilizing an FEI Titan operated at 80 kV and 300 kV. Small drops of the zinc nanoparticle water suspension were deposited onto a QUANTIFOIL® Holey Carbon Film on copper TEM grids. Widths of the interference fringes were measured and the Miller-Bravais indices of the crystalline structures were estimated by the Crystallography lab software [22].

### AFM

Metal nanoparticles were imaged by Bruker MultiMode 8 (Santa Barbara, CA) atomic force microscope in Tapping® (intermittent-contact) mode. Futher details of AFM measurements are described in [23].

### XPS

XPS was utilized to measure atomic composition of the nanoparticles’ surfaces. The Kratos Axis Ultra delayline detector (DLD) instrument in the hybrid mode used a monochromatic Al Kα1, 2 X-ray source (hυ = 1486.6 eV) as described in [23]. The ratio of the Zn and ZnO were obtained from the high-resolution spectra of Zn 2p (1017 eV to 1057 eV) and were measured with the energy resolution of 0.1 eV. Water suspensions of the nanoparticles were spread onto the gold-coated silicon wafers, and evaporated during evacuation of the system.

### Zeta potential

Zeta potentials of the nanoparticles in water suspension were measured with a Zetasizer Nano ZSP (Malvern Instruments, Worcestershire, UK) by the LDV technique [23]. The average of six sequential runs were obtained. Zeta potentials were estimated by Henry’s equation:

ζ=3ημ/2εF(kα)

in which ζ is the zeta potential, η is the viscosity, μ is the electrophoretic mobility, ε is the dielectric constant of the medium and F(*kα)* is Henry’s function, which equals 1.5 using the Smoluchowski equation [24].

### Odorants

Odorants were obtained from Sigma-Aldrich. An odorant mixture of 1.6 mmole/L each of ethyl butyrate, eugenol, and (+) and (–) carvone in water was prepared with a vortex mixer and stored in a dark glass container at 283K (5 ^o^C).

### Delivery of odorants and metal nanoparticles

During experiment, a 0.25 s pulse of the odorant mixture at 55158 N/m^2^ (8 psi) was generated by a computer-controlled Pneumatic PicoPump PV800 (World Precision Instruments, Sarasota, FL). A pulse of positive pressure pushed the odorant into a glass nozzle toward the OE. The computer controlled odorant delivery was comprised of 0.25 s pulses at 20 s and 60 s times for EOG data collection. The single EOG recording took 200 s and contained 10 response traces. A nanoparticle suspension was combined with odorant solutions to reach a final nanoparticle concentration of 0.02 nmole/L. During the pulse of delivery, the vapor of odorant with metal nanoparticles was transferred to the OE surface [2].

### Animals

The animal protocol was approved by the Auburn University Institutional Animal Care and Use Committee (AU IACUC). Adult male Sprague–Dawley rats (Envigo, Dublin, VA) weighing ~300 g were used. EOG olfactory experiments over a storage period of 317 days were carried out with different animals as described in [2, 23, 25].

### Electrophysiology

We used electroolfactography (EOG) [26]. The instrumentation included Axon Instrument MultiClamp 700A amplifier and 1322A DigiData acquisition system. Rat OE was dissected out and positioned in a perfusion chamber such that the basal parts were immersed in physiological solution, while the olfactory cilia ware positioned in the water/air interface. Glass electrodes of ≈ 24 μm tip opening were linked to the amplifier to record signals from the OE. After connection between the electrode and the OE was made, air pulse of the odorant mixture was applied and a continuous EOG signal was recorded as function of time. All olfactory experiments were performed *ex vivo*. The primarily-elemental or PEGylated zinc nanoparticles were delivered with the odorant mixture. The relative enhancement of olfactory responses by zinc nanoparticles were calculated as (EOG_O+Zn_-EOG_O_)/EOG_O_, where EOGo was the peak of electrical response evoked by the odorant alone, and EOG_O+Zn_ was the peak response stimulated by the mixture of the odorant and Zn nanoparticles. Similarly, the relative enhancements by ZnPEG400 and ZnPEG1000 were determined as (EOG_O+ZnPEG400_-EOG_O_)/EOG_O_ and (EOG_O+ZnPEG1000_-EOG_O_)/EOG_O_, respectively.

Further details of the method were described in [2, 23].

### Statistical analysis

Data averaging, ANOVA, t-test, curve fitting, and graph plotting were carried out using Origin 2015 (Northampton, MA) and 2010 Microsoft Excel.

## Results

### Characterization of PEGylated zinc nanoparticles

#### Properties of PEG on the surface of zinc nanoparticles

Zinc nanoparticles were engineered from metal bars by an electrolysis method [4] and were PEGylated with 400 g/mol or 1000 g/mol molecular weight polyethylene glycol (S1 Supporting Information), and referred hereafter as ZnPEG400 and ZnPEG1000, respectively. The properties of the PEG molecules on the nanoparticle surface are shown in Table 1. PEG400, with 14 fewer monomers than PEG1000, was predicted to form a thinner coating on the surface of zinc nanoparticles (S1 Supporting Information).

**Table 1:** Properties of PEG on the surface of zinc nanoparticles.

Property	ZnPEG400	ZnPEG1000	Reference
**Area per PEG single chain, nm^2^**	1.3	2.91	[27]
**Number of monomers in PEG**	9	23	S1 Supporting Information
**Thickness of PEG layer, nm**	1.3	2.3	S1 Supporting Information

#### AFM

**Fig 1:**
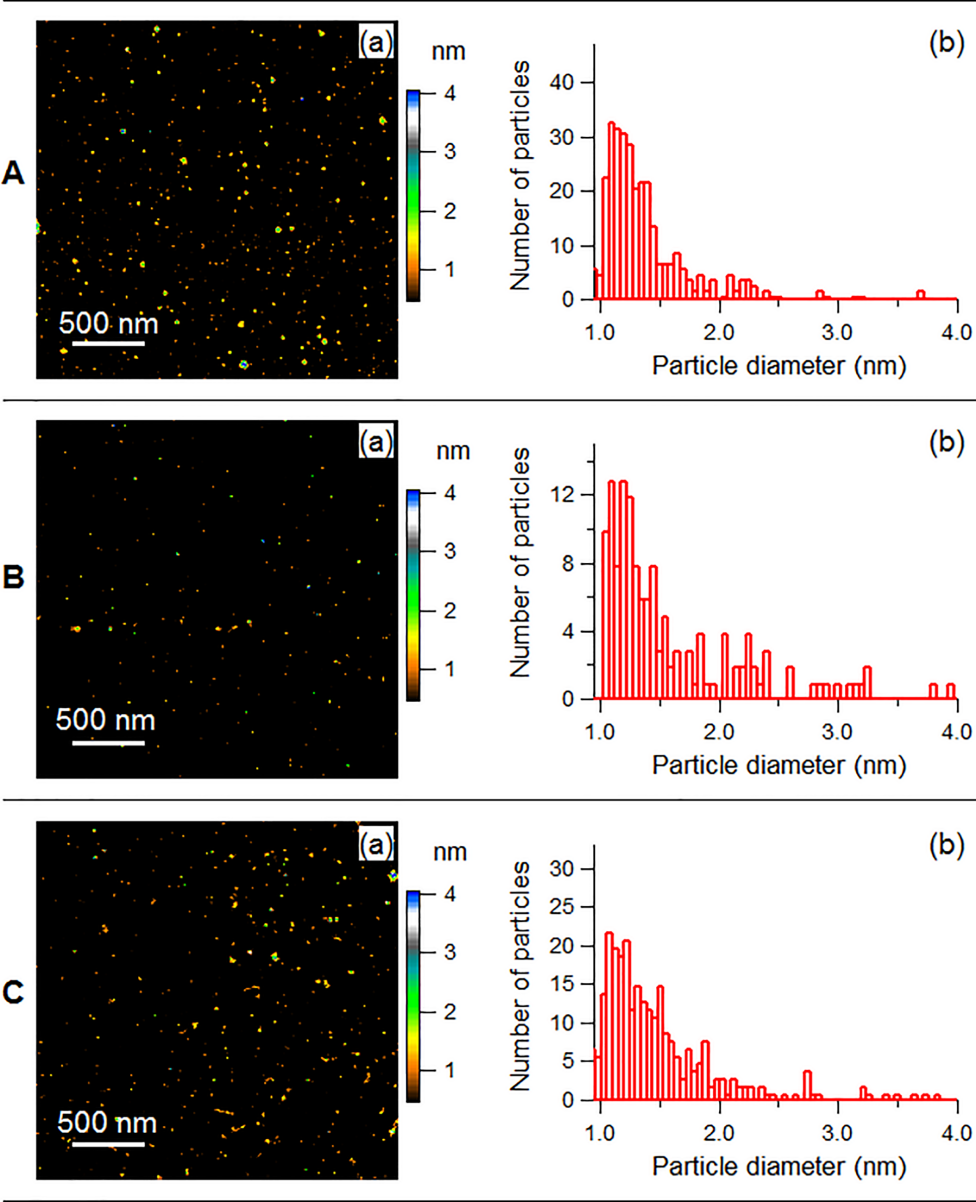
Physical properties of non-PEGylated and PEGylated zinc nanoparticles. (a) AFM images, and (b) histograms showing the size distribution of the nanoparticles. A: Zinc nanoparticles, B: ZnPEG400, C: ZnPEG1000.

Fig 1 demonstrates the physical properties of non-PEGylated and PEGylated zinc nanoparticles. AFM showed the diameter distributions of zinc nanoparticles, with mean sizes of 1.2 ± 0.3 (SD) nm (Fig 1A, a and b). Fig 1A, b shows the histogram with a peak around the mean value. Similarly, the size distributions of ZnPEG400 and ZnPEG1000 revealed the average diameter of 1.4±0.4 (SD) nm for both formulations. (Fig 1B, b and 1C, b).

#### TEM

**Fig 2:**
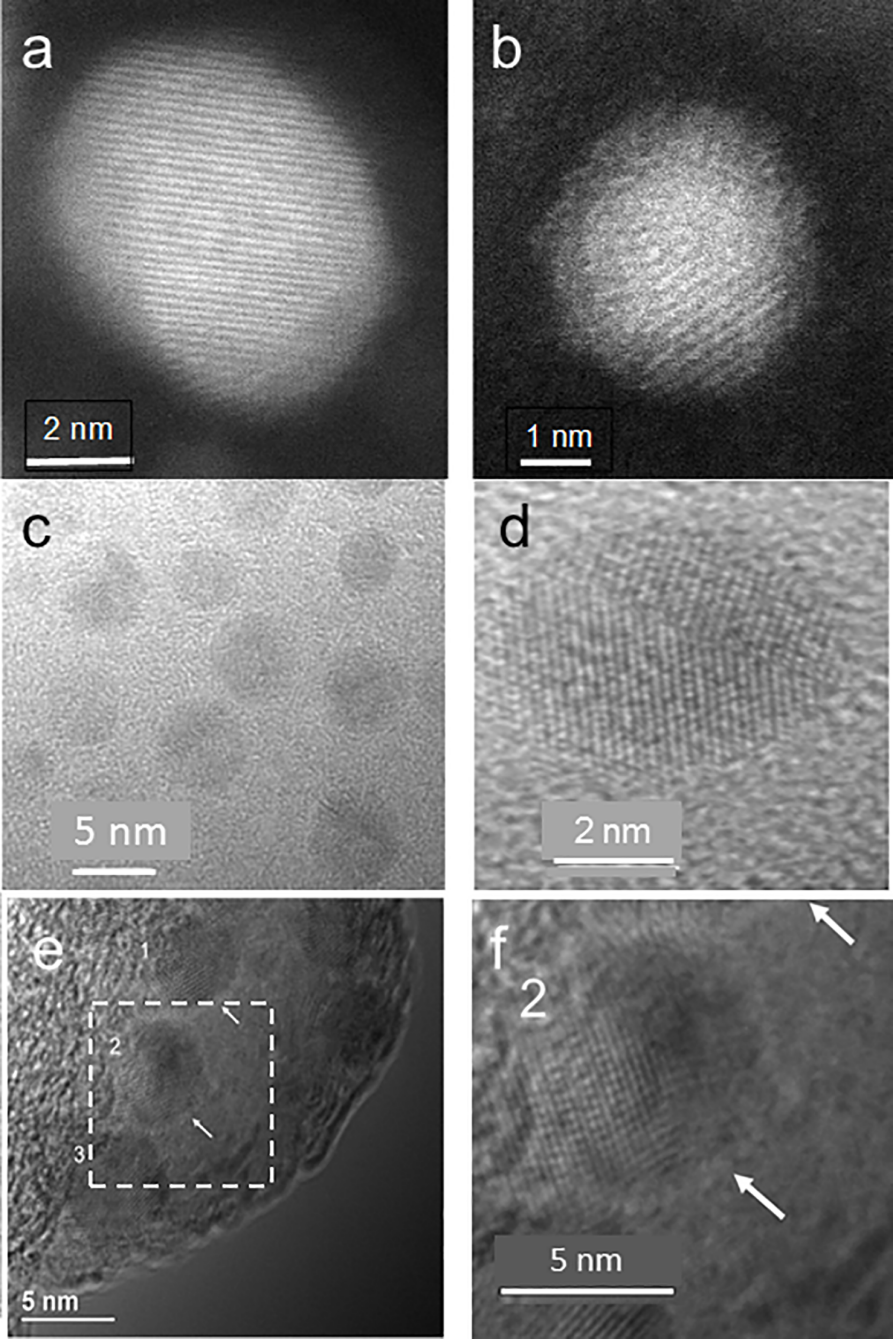
Electron microscopy images of Zn, ZnPEG400, and ZnPEG1000 nanoparticles. TEM images (a, c, d) are showing the primarily-elemental zinc nanoparticles at different magnifications, (b) ZnPEG400 nanoparticle showing the metal core and the PEG passivation layer, (e) ZnPEG1000 nanoparticles with negative staining, and (f) ZnPEG1000 magnified from (e). The numbers 1, 2, 3 point to the nanoparticles with visible lattice fringe patterns, indicating their crystallinity.

Fig 2 summarizes the electron microscopy images of Zn, ZnPEG400, and ZnPEG1000 nanoparticles. Panels a and b show TEM micrographs of Zn and ZnPEG400 nanoparticles, respectively. Both particles show lattice fringes. Panels c and d provide TEM images of zinc nanoparticles also showing their crystalline character. Panel e illustrates TEM image of ZnPEG1000 nanoparticles. Arrows show layers surrounding nanoparticles that are presumed to be coatings of PEG1000. Panel f depicts the enlarged dashed square shown in panel e. Within the uncertainty of our data accuracy, we determined that the characteristic fringes of 0.21 nm and 0.17 nm found in both non-PEGylated and PEGylated zinc nanoparticles match the (011) and (012) vectors for the hcp crystal lattice of zinc. Larger than average size particles are shown to emphasize the crystal fringes.

#### XPS

The non-PEGylated and PEGylated zinc nanoparticles were subjected to four experimental conditions: 1. Freshly prepared nanoparticles stored for 1 day at of 278 K (5 ^o^C); 2. Particles stored for 317 days at 278 K (5 ^o^C); 3. Particles stored for 2 days at the temperature of 303 K (30 ^o^C); and 4. Particles stored for 2 days at 323 K (50 ^o^C). Physical analyses of all the samples were performed immediately after the end of each of the exposure conditions to minimize any changes in chemistry.

**Fig 3:**
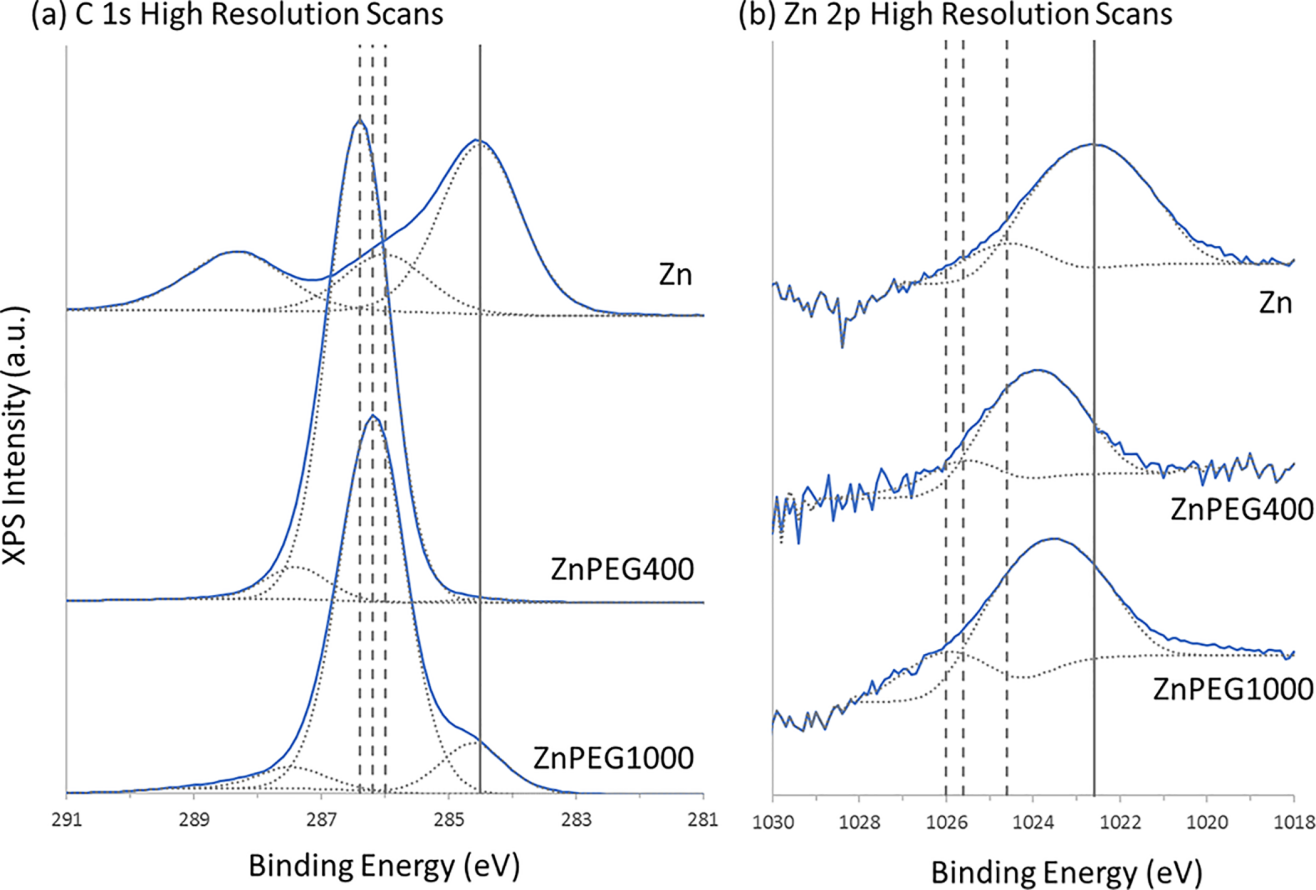
XPS spectra. Representative high resolution XPS spectra showing the (a) C 1s and (b) Zn 2p3/2 core lines for the freshly prepared bare zinc and PEGylated zinc nanoparticles stored 1 day at 278 K (5 ^o^C), with the spectra offset to facilitate viewing. The Zn 2p spectra are shown in log-scale. The solid curves indicate the experimentally obtained spectra, with the dotted curves underneath indicating their best-fit chemical components. For the C 1s spectra, from lower to higher binding energy, the components are C-C, C-O, and C = O. For the Zn 2p spectra, the components are Zn and ZnO. For the (a) C 1s plots, the solid vertical line represents the position of the C-C peaks, to which all spectra were calibrated to, with the dotted lines showing the spectral shift of the C-O peaks, which were 286 eV, 286.4 eV, and 286.2 eV for the Zn, ZnPEG400, and ZnPEG1000 systems, respectively. For (b) Zn 2p plots, the solid line represents the position of the Zn peak for the bare Zn system, while the dotted lines show the spectral shift of the ZnO peaks, which were 1024.6 eV, 1025.6 eV, and 1026.0 eV for the Zn, ZnPEG400, and ZnPEG1000 systems, respectively. Each spectrum represents an average of six spectral runs.

Fig 3 shows the representative XPS spectra obtained for freshly prepared zinc and PEGylated zinc nanoparticles stored 1 day at 278 K (5 ^o^C). A summary of the XPS data for non-PEGylated and PEGylated zinc nanoparticles for all four experimental conditions is shown in Table 2. The spectra were calibrated using the adventitious carbon C 1s peak at a binding energy of 284.6 eV, which allowed the identification of the following chemical species at their respective binding energies: hydrocarbon (C-C≈285 eV), ether (C-O, ≈286.1), carboxyl (C = (OH), ≈288.1) [28], zinc (Zn 2p_3/2_, ≈1021.8 eV) [29, 30] and ZnO (Zn 2p_3/2_, ≈1024 eV) [31]. The rise in the amplitude of C-O peak and the reduction in the binding energy of Zn 2p_3/2_ peaks in the ZnPEG400 and PEG1000 samples confirmed the successful PEGylation of zinc nanoparticles. The Zn 2p_3/2_ band shown that metallic zinc nanoparticles were oxidized only slightly, where the concentrations of Zn and ZnO were found to be 97.0 ± 0.8 (SD) % and 3.0 ± 0.8 (SD) %, respectively (Table 2, 278 K, 1 day). Interestingly, the PEGylation process caused a significant increase in the concentration of oxidized zinc atoms, where the interaction with PEG400 (278 K, 1 day) resulted in an increase of ZnO concentration from 3.0±0.8 (SD) % to 10.5±4.0% (t(5) = 4.93, p = 0.004), while in the case of PEG1000, the ZnO concentration increased to 12.0±3.8% (t(5) = 6.47, p = 0.001).

**Table 2:** XPS properties of bare and PEGylated zinc nanoparticles, stored at various durations and temperatures.

Sample	T K	Time days	Survey			High Resolution Zn 2p				High Resolution C 1s					
			O1s %	C 1s %	Zn2p %	BE eV	Zn %	BE eV	ZnO %	BE eV	C-C %	BE eV	C-O %	BE eV	C = O(OH) %
**Zn**	278	1	27.8 ± 2.3	70.5 ± 1.7	1.8 ± 0.1	**1021.8**	**97.0 ± 0.8**	**1024.6**	**3.0 ± 0.8**	284.3	66.2 ± 2.9	286.2	22.6 ± 1.5	288.5	11.2 ± 1.7
**ZnPEG400**	278	1	32.3 ± 0.5	67.4 ±0.7	0.3 ± 0.1	**1022.0**	**89.5 ± 4.0**	**1023.8**	**10.5 ± 4.0**	285.0	3.7 ± 2.1	286.9	92.0 ± 3.0	288.5	4.3 ± 1.0
**ZnPEG1000**	278	1	30.3 ± 0.9	69.6 ± 2.1	0.1 ± 0.0	**1021.9**	**88.0 ± 3.8**	**1023.5**	**12.0 ± 3.8**	283.2	3.0 ± 0.6	286.7	94.5 ± 0.6	290.2	2.6 ± 0.0
**Zn**	278	317	38.4 ± 1.0	60.2 ± 1.5	1.4 ± 0.1	**1021.9**	**96.0 ± 1.0**	**1023.7**	**4.0 ± 1.0**	284.4	60.2 ± 0.9	285.8	18.6 ± 0.7	288.1	21.2 ± 0.5
**ZnPEG400**	278	317	31.5 ± 0.1	68.4 ± 0.1	0.1 ± 0.0	**1022.2**	**75.9 ± 8.2**	**1023.9**	**24.1 ± 8.2**	285.0	0.9 ± 0.2	287.0	92.8 ± 0.9	288.0	6.3 ± 0.8
**ZnPEG1000**	278	317	31.1 ± 0.3	68.0 ± 0.4	0.9 ± 0.2	**1021.9**	**86.1 ± 0.5**	**1024.2**	**13.9 ± 0.5**	285.1	11.5 ± 2.4	286.7	83.5 ± 1.9	288.0	5.0 ± 0.8
**Zn**	303	2	24.5 ± 4.1	75.1 ± 0.6	0.4 ± 0.1	**1021.7**	**92.2 ± 3.5**	**1024.1**	**7.8 ± 3.5**	284.4	60.8 ± 6.8	286.5	31.5 ± 4.0	289.2	7.7 ± 4.0
**ZnPEG400**	303	2	23.2 ± 4.4	76.6 ± 3.0	0.2 ± 0.0	**1022.0**	**93.0 ± 2.8**	**1023.7**	**7.0 ± 2.8**	284.4	38.9 ± 15.0	286.8	56.9 ± 15.3	288.8	4.2 ± 0.5
**ZnPEG1000**	303	2	24.6 ± 5.4	75.2 ± 5.2	0.2 ± 0.1	**1021.8**	**94.2 ± 5.6**	**1023.4**	**5.8 ± 5.6**	284.4	39.2 ± 14.9	286.6	55.7 ± 14.9	288.6	5.1 ± 1.1
**Zn**	323	2	38.3 ± 5.3	60.8 ± 3.5	0.9 ± 0.3	**1021.0**	**92.9 ± 3.3**	**1023.3**	**7.1 ± 3.3**	284.2	65.6 ± 14.7	285.9	20.6 ± 8.8	288.2	13.8 ± 6.1
**ZnPEG400**	323	2	27.7 ± 4.6	72.1 ± 6.8	0.2 ± 0.1	**1021.9**	**88.6 ± 2.6**	**1023.7**	**11.4 ± 2.6**	284.4	35.2 ± 10.7	286.9	62.6 ± 12.7	283.7	2.2 ± 0.1
**ZnPEG1000**	323	2	21.8 ± 3.5	77.9 ± 2.7	0.3 ± 0.1	**1021.7**	**92.7±1.6**	**1024.0**	**7.3±1.6**	284.4	50.5 ± 15.7	286.5	43.7 ± 15.4	288.6	5.8 ± 0.8

BE refers to the binding energy of the peak, and % refers to the relative atomic composition of the elements normalized to the elements shown below, within their respective scans (*i*.*e*., Survey, High Resolution Zn 2p and C 1s).

The XPS survey scan of the freshly prepared non-PEGylated and PEGylated zinc nanoparticles (Table 2, 278 K, 1 day) showed the concentration of the O 1s and C 1s species to be 27.8±2.3% and 70.5±1.7%, respectively, which significantly changed to 32.3±0.5%(t(5) = 4.28, p = 0.003) and 67.4±0.7% (t(5) = 3.77, p = 0.005) for ZnPEG400, respectively. The values of O 1s and C 1s for ZnPEG1000 were 30.3±0.9% (t(5) = 2.26, p = 0.0053) and 69.6±2.1% (t(5) = 0.74, p = 0.48), respectively (there were no statistically significant changes for ZnPEG1000).

ZnPEG400 after 317 days of storage, showed the higher level of ZnO of 24.1±8.2% ZnO compared to 10.5±4.0% (t(5) = 3.45, p = 0.008) for the freshly prepared ZnPEG400, as shown in Table 2.

Very different XPS results were observed for ZnPEG1000 after storage. The concentration C-O group showed a modest decrease from 94.5±0.6% to 83.5±1.9% (t(5) = 12.3, p = 0.0001), but a sensible increase in the C-C concentration from 3.0±0.6% to 11.5±2.4% (t(5) = 7.68, p = 0.0001). The concentration of ZnO for this condition were 12.0±3.8% and 13.9±0.5% (t(5) = 1.11, p = 0.3) showed no significant difference. These data suggest no significant changes in the electronic structure of both Zn and PEG1000 after the initial PEGylation.

#### Arrhenius activation energies of oxidation

Oxidation of the zinc nanoparticles was observed under storage at different temperatures and duration, as evidenced by the increase of ZnO components in Table 2. Using an apparent concentration of primarily- elemental zinc as a function of time and storage temperature, the Arrhenius activation energies of oxidation were estimated [32] by equation (S6) (S1 Supporting Information) and shown in Table 3.

**Table 3:** Thermodynamic analysis of oxidation of Zn, ZnPEG400, and ZnPEG1000 nanoparticles.

Particle	T ^o^C	Estimated activation energy, E_a_ kJ.mol^-1^	Reference
**Zn, 1.2 nm**	5–50	113	Present work
**Zn foil, 0.126 mm**	300–400	119	[33]
**ZnO powder, oxygen desorption**	86–97	96	[34]
**Molten Zn**	600–700	104	[35]
**ZnPEG400, 1.4 nm**	5–50	15.5	Present work
**ZnPEG1000, 1.4 nm**	5–50	34.9	Present work
**Pd/Fe nanoparticles, 60–100 nm**	20–35	39.47	[36]
**Pd/FePEG200**	20–35	38.66	[36]

#### Zeta potential

The change in the physical properties of zinc nanoparticles after PEGylation is also confirmed by alteration of the zeta potential. The PEG binding resulted in a sharp reduction of zeta potential in water. Zeta potential showed values of -42.4 ± 4.8 (SE) mV, -26.1 ± 2.5 (SE) mV (t(7) = 7.96, p = 0.0001), and -27.5 ± 2.5 (SE) mV (t(7) = 7.28, p = 0.0001) for Zn, ZnPEG400, and ZnPEG1000 nanoparticles, respectively.

### Enhancement of odorant response by PEGylated nanoparticles

**Fig 4:**
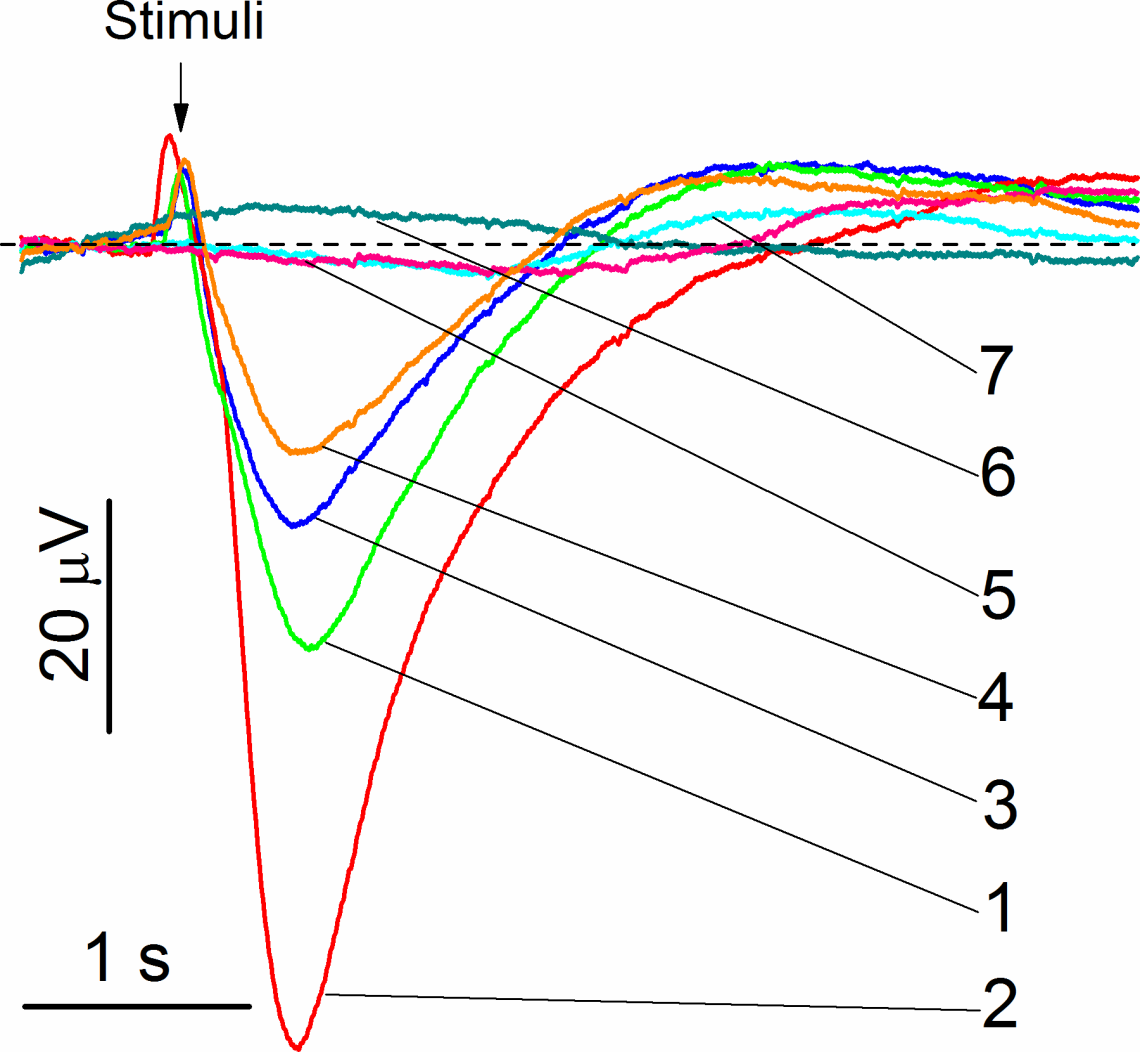
Representative EOG recordings from rat olfactory epithelium. a The stimuli were of 0.25 s pulses of (1) odorant mixture, (2) odorant mixture +1.2 nm zinc nanoparticles, (3) odorant mixture + 0.25 mmole/L PEG400, (4) odorant mixture + 0.10 mmole/L PEG1000, (5) water vapor + PEG400, (6) water vapor + PEG1000, and (7) water vapor. The concentration of zinc nanoparticles and odorant mixture were 0.02 nmole/L and 1.6 mmole/L, respectively. The representative set of traces was obtained from 50 EOG traces.

The initial experiments were carried out with the freshly prepared non-PEGylated and PEGylated zinc nanoparticles. Representative electroolfactograms showing effects of PEG400 and PEG1000 without zinc nanoparticles and recorded from the rat OE are shown in Fig 4. The EOG responses evoked by PEG400 and PEG1000 without odorant mixture are very small and hardly can be distinguished from the EOG signal of water. This result is consistent with polyethylene glycol being odorless [37]. The EOG evoked by the odorant mixture with zinc nanoparticles (trace 2) shows a strong enhancement compared to the signal induced by the odorant mixture alone (trace 1). Combining odorants with ZnPEG400 and ZnPEG1000 results in the lower signals depicted by the traces 3 and 4, respectively. The mean values of seven measurements of the relative responses to the odorant mixtures with Zn, ZnPEG400, and ZnPEG1000 were 2.69±0.11, 0.821±0.05, and 0.310±0.04, respectively. An analysis of variance showed that at the 0.05 level, the means were significantly different, F(2, 18) = 1900, p = 0.000, indicating that the freshly prepared Zn nanoparticles were superior to ZnPEG400 and ZnPEG1000, and ZnPEG400 with odorant produced larger relative responses than ZnPEG1000 with odorant.

The olfactory responses to odorant mixed with PEG400 or PEG1000 were tested systematically over the period of 317 days. As evidenced by the foregoing data, the freshly prepared Zn nanoparticles outperformed both ZnPEG400 and ZnPEG1000. However, as analysis of variance showed, at the level of 0.05, there was no significant difference between the relative olfactory responses to Zn, ZnPEG400, and ZnPEG1000 nanoparticles ranging from 3 days to 64 days of storage (F_3 days_(2, 25) = 0.64, p = 0.54; F_5 days_(2, 69) = 2.53, p = 0.087; F_18 days_(2, 30) = 0.92, p = 0.41); F_58 days_(2, 12) = 1.14, p = 0.35; and F_64 days_ = (2, 21) = 3.12, p = 0.065). Only after 280 days and 317 days of storage we observed the significant difference between the relative olfactory responses to Zn, ZnPEG400, and ZnPEG1000 nanoparticles (F_280 days_(2, 48) = 90, p = 0.000; F_317 days_(2, 36) = 149, p = 0.000).

**Fig 5:**
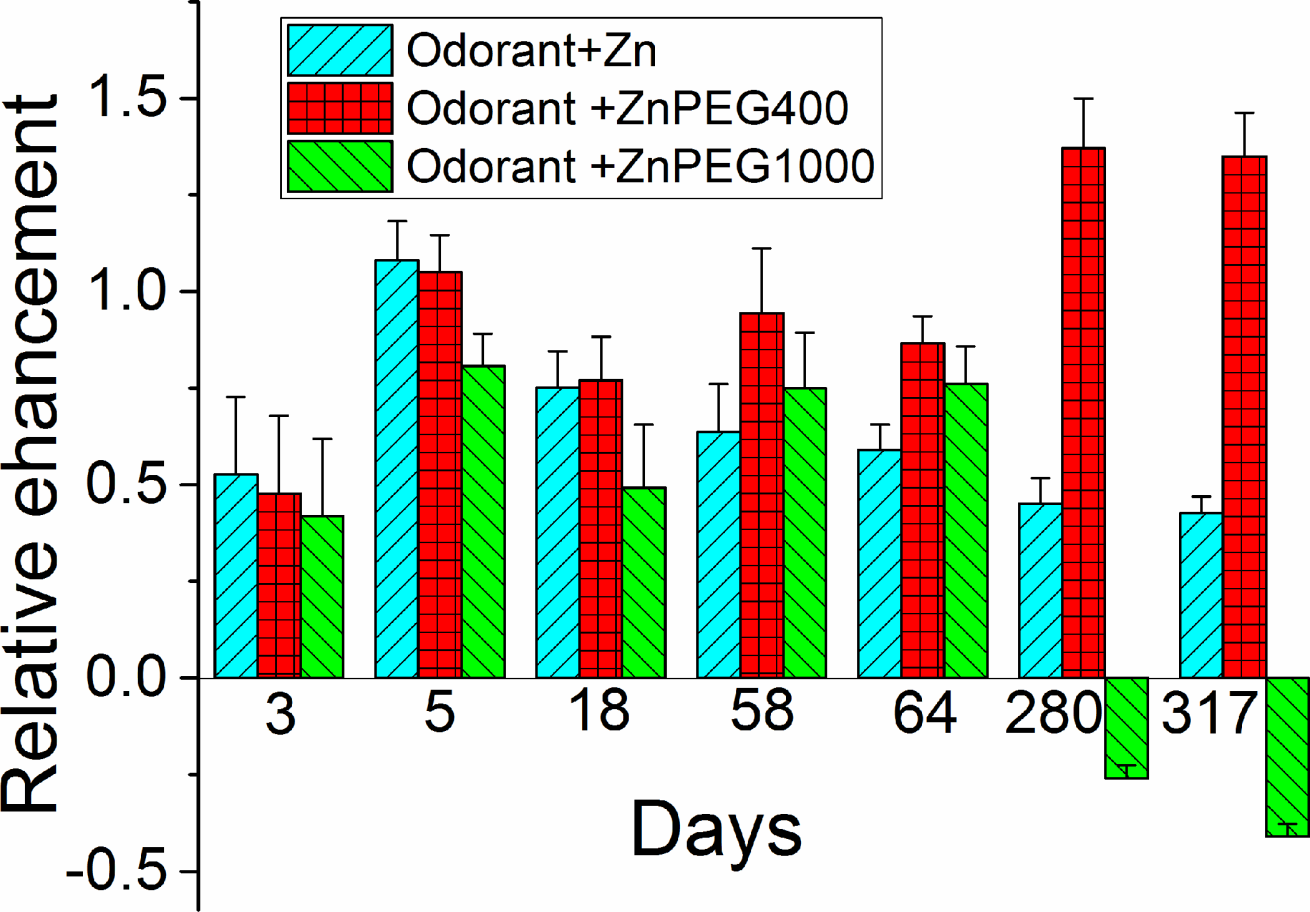
The relative EOG signals as a function of time of storage of zinc nanoparticles. The difference between peak values of EOG evoked by odorant and by non-PEGylated and PEGylated zinc nanoparticles was normalized by the EOG peak evoked by an odorant alone. The relative enhancement by zinc nanoparticles was calculated as described in Materials and methods.

Following 280 days of storage, ZnPEG400 provided the highest enhancement followed by Zn and then ZnPEG1000. Near the end of the storage time, the mixture odorant and ZnPEG1000 caused a decrease of the EOG signal compared to the signal generated by the odorant alone (Fig 5).

**Fig 6:**
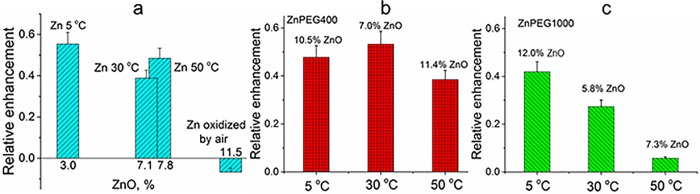
The relative EOG signals evoked by non-PEGylated and PEGylated zinc nanoparticles. **a**. The relative enhancement by non-PEGylated zinc nanoparticles as a function of ZnO concentration. The difference between peak values of EOG evoked by odorant and by zinc nanoparticles was normalized by the EOG peak evoked by an odorant alone as a function of ZnO concentration that was determined by XPS. The first bar at 3% of ZnO, corresponds to zinc nanoparticles stored one day at 278 K (5 ^o^C). The second and third bars reflect zinc nanoparticles stored for two days at 303 K (30 ^o^C) and 323 K (50 ^o^C), at 7.1% and 7.8% respectively. The forth bar at 11.5% of ZnO corresponds to the negative enhancement (inhibition) that was observed with zinc nanoparticles oxidized by percolating air. **b**. The relative EOG enhancement produced by ZnPEG400 nanoparticles after they were stored for one day at 278 K (5 ^o^C), two days at 303 K (30 ^o^C) and 323 K (50 ^o^C), respectively. **c**. The relative EOG enhancement produced by ZnPEG1000 nanoparticles after they were stored for one day at 278 K (5 ^o^C), two days at 303 K (30 ^o^C) and 323 K (50 ^o^C), respectively.

To characterize effects of thermally-enhanced formation of ZnO on the odorant responses, we analyzed the effects of non-PEGylated and PEGylated zinc nanoparticles stored for two days at an elevated temperature. Fig 6 shows the relative enhancement of responses to odorants as the function ZnO concentration determined by XPS and temperature. The relative olfactory enhancement by the zinc nanoparticles declined sharply from 55% to -7%, when the ZnO concentration increased from 3.0% to 11.5% (Fig 6A). The enhancement with ZnPEG1000 did not show the obvious dependence on the ZnO concentration but it strongly declined with the temperature (Fig 6C). The enhancement with ZnPEG400 (Fig 6B) appeared not to be very sensitive to zinc oxidation and temperature.

## Discussion

### Characterization of PEGylated zinc nanoparticles

The crystalline structure of zinc nanoparticles revealed in this work is consistent with those obtained for zinc nanowires and nanorods [38, 39]. The equivalence of the zinc lattices and core lattices of PEGylated zinc nanoparticles indicates that PEGylating maintains the crystalline nature of the metal particles. To further analyze physical properties of the non-PEGylated and PEGylated zinc nanoparticles, XPS was utilized for study of nanoparticles in the variety of experimental conditions.

The decrease in the binding energy of Zn 2p_3/2_ peaks in the ZnPEG400 and PEG1000 nanoparticles and the rise in the amplitude of the XPS C-O peak indicated that PEGylation of zinc nanoparticles was successful.

The Zn 2p_3/2_ band shown that metallic zinc nanoparticles were oxidized only slightly, where the concentrations of Zn and ZnO were found to be 97.0 ± 0.8 (SD) % and 3.0 ± 0.8 (SD) %, respectively (Table 2, 278 K, 1 day).

The interaction of the PEG molecules and zinc nanoparticles had a remarkable effect on the binding energies of carbon and zinc. Bonding of the molecules onto the nanoparticles was associated with an increase in the binding energy of the C-O bond, and a corresponding decrease in the ZnO binding energy. For the nanoparticles held at 278 K for 1 day (Table 2), for example, the C-O binding energy increased from 286.2 eV to 286.9 eV (by roughly 0.7 eV) while the ZnO binding energy decreased by 0.9 eV when the nanoparticles were covered by PEG400. A similar shift in binding energies were observed for the nanoparticles covered in PEG1000. We speculate that the PEG molecules are interacting with the zinc atoms in such a way that the electronic structure of the nanoparticles is affected, and a conjugated electron system is produced [40]. It is likely that long polymer chains increase the withdrawal of electron charge and electronic stability, which subsequently increases the binding energy of the C and O elements within the chain [41]. Conversely, the polymer chains may act as a passivation layer that leads to a reduction in binding energy because of the fact that the zinc nanoparticles are acting as electron donors, which has been observed for platinum nanoparticles below 7 nm in size [42].

The highest shift in binding energy was observed for ZnPEG400 nanoparticles stored at 278 K for 317 days (Table 2). The binding energy of the C-O component increased by 0.9 eV relative to the control value of 286.2 eV, and a corresponding decrease in binding energy of 0.7 eV was seen for ZnO. Similar shifts in binding energy were also observed for PEGylated zinc nanoparticles exposed to elevated temperatures of 303 K (30 ^o^C) and 323 K (50 ^o^C). These observations point to the covalent binding of PEG to the engineered zinc nanoparticles, with similar binding energy shifts observed after PEGylation of MoS_2_ nanoflakes [43, 44], gadolinium oxide thin film and nanoparticles [45], and gold nanorods [46].

The value of the activation energy for the oxidation of zinc nanoparticles agrees well with that obtained by the oxidation of zinc foil [33], the oxygen desorption from ZnO powder, and the oxidation of molten zinc [35]. After PEGylation, zinc nanoparticles showed reduction of the activation energy, with the binding of PEG400 producing a larger energy reduction compared to PEG1000. No literature was available for comparison of the activation energy of the PEGylated zinc nanoparticles. However, a decrease in the activation energy was reported when 60–100 nm Pd/Fe nanoparticles underwent PEGylation [36].

The reduction of the nanoparticle surface charge after PEGylation may explain an increased affinity to target proteins [5].

### Enhancement of odorant response by PEGylated nanoparticles

Previously, we showed that primarily-elemental zinc nanoparticles strongly enhanced responses to odorants, while zinc nanoparticles oxidized by air did not enhance but slightly inhibited the responses (11.5% ZnO compared to 3.0% for the primarily-elemental nanoparticle) [4]. The olfactory enhancement of approximately 140% using ZnPEG400 after 317 days of storage was not an anticipated result based on the higher level of ZnO of 24.1% ZnO compared to 10.5% for the freshly prepared ZnPEG400, as shown in Table 2. Although no significant change in the C-O concentration was observed, the C-C concentration decreased from 3.7% to 0.9%. The increase in the observed level of ZnO in this case does not imply a high level of oxidized zinc atoms, but rather seems to reveal that there is a formation of a unique conjugated electron system between the zinc nanoparticles and PEG400 molecules that may be responsible for the increase in olfactory response.

To explain the mechanism of enhancement and inhibition by ZnPEG400 and ZnPEG1000, respectively, it is essential to consider what has been discovered about the olfactory enhancement by zinc nanoparticles.

We have reported that two molecules of olfactory receptor binds a single zinc nanoparticle to create a dimer [47]. We suggested that the odorant cannot activate a single receptor.

When two receptors bind to an endogenous zinc nanoparticle, the created dimer participates in signal trunsduction. The remaining receptors are monomeric [48]. Consequently, the dimerization mechanism enabled by the zinc nanoparticles provides an explanation for the olfactory enhancement. When the OE is exposed to a mix of the odorant and engineered zinc nanoparticles, the nanoparticles link previously unconnected receptors and create new dimers. The enhancement of olfactory response is therefore explained by the contribution of the created dimers in the signal transduction.The olfactory enhancement by zinc nanoparticles is significant in consideration of Luca Turin’s vibrational hypothesis that proposed an inelastic electron tunneling spectroscopy (IETS) mechanism for discriminating odorants [49]. The model needs a donor of electrons, and it is rational to propose that elemental zinc in the nanoparticles become electron donors [3, 4, 47, 50]. Therefore, zinc nanoparticles play a dual role in the enhancement of olfaction: they increase the concentration of receptor dimers and serve as electron donors for the tunnel electron transport.

The addition of the ZnPEG400 nanoparticles to odorant at the end of 317 days of storage at 278 K (5 ^o^C) resulted in the highest relative EOG enhancement that was larger than enhancement by the un-PEGylated zinc nanoparticles. This amplified enhancement can be explained by the increased binding affinity to olfactory receptors in part due to the reduction of the surface charge as it was manifested by the reduction of zeta potential and because of the increase of hydrophobicity [51]. Additionally, XPS data indicate covalent binding between PEG400 and zinc nanoparticles that may cause oxidative coupling between the PEG molecules and zinc atoms on the surface of zinc nanoparticles. The combined electron system may be more efficient in donating electrons than non-PEGylated zinc nanoparticles and therefore contributed to the amplified olfactory enhancement. The signal amplification effects for combined electron system was reported for PEGylated electrodes [52–55]. Furthermore, the estimated transmission probability of electron transfer through the PEG400 layer was larger than that for the PEG1000 (S1 Supporting Information). All the above properties of ZnPEG400 (increased binding affinity, combined zinc-PEG electron system, and favorable electron transmission probability) provide an explanation for the amplified olfactory enhancement compared to non-PEGylated zinc nanoparticles.

The XPS analysis of the ZnPEG1000 after long storage indicated that in contrast to ZnPEG400 data, zinc nanoparticles and PEG1000 did not produce a conjugated electron system, and therefore they do not have the advantage of facile electron donation compared to non-PEGylated zinc. This observation may be explained by the length, viscosity, and rigidity of PEGs. PEG400 with ~9 monomer repeats (as opposed to ~23 repeats in PEG1000) has a lower viscosity and rigidity than those of PEG1000 [56]. With the lower viscosity and rigidity, the PEG 400 can enable a more intimate interaction with the olfactory sensory receptors. Moreover, being a shorter molecule the PEG400 produced a thinner layer around the zinc nanoparticle. The estimated transmission probability of electron transfer through the PEG400 layer was approximately 4 times higher than that of the PEG1000 (S1 Supporting Information). The PEG400 layer, therefore, can provide the sufficient electron transfer for the olfactory transaction, while PEG1000 is much less efficient. ZnPEG1000 nanoparticles showed negative relative EOG signals post 280 days incubation because they compete with the endogenous zinc nanoparticles for binding sites in the olfactory receptors. The ZnPEG1000 nanoparticles occupied the receptor binding sites that were intended for endogenous zinc nanoparticles. This binding resulted in the receptor dimers that were not capable of triggering the signal transaction because zinc nanoparticles could not transfer electrons through the PEG1000 layers. Thus, the produced signal evoked by the odorant with ZnPEG1000 nanoparticles will be smaller than the signal evoked by the odorant alone (Fig 5).

The relative enhancement of zinc nanoparticles had a non-monotonic dependence on the time of storage. The freshly prepared particles had the highest enhancement. The enhancement declined on day 3 and then increased again on day 5 (Fig 5). This non-monotonic behavior cannot be explained through the animal to animal variability. The amplitude of the electrical signals varies from cell to cell and from animal to animal [57]. The EOG measured at the different contact points of OE may have a different amplitude. However, the earlier measurements showed that the correctly normalized relative values of the epithelial electrical signals were conserved amongst the single animal and different animals of the same age and breed [58, 59]. This fact allowed the use of different animals to quantitatively characterize initial olfactory events. We previously used this approach to characterize the effects of different odorants at various concentrations and evaluate the effects of zinc nanoparticles [2]. We further showed that the cultured olfactory neurons initially obtained from different mouse pups also conserved the EOG kinetic properties of the neurons at the same age. For these cultured neurons, we also showed that zinc nanoparticles enhanced EOG responses of both younger and older cultures [25].

We speculate that non-monotonic dependence of an olfactory enhancement by zinc nanoparticles can be attributed to the time-dependent changes in the electronic structure of the nanoparticles related to the relaxation and diffusion of atoms. Indeed, taking into the account that the self-diffusion coefficient of zinc in the single zinc crystal at 278 K is 7.7×10^−22^ m^2^/s [60], it would take ≈31 h to move a zinc atom the distance of 1.2 nm. This time is consistent with the time of the initial decline in the relative olfactory enhancement. Zinc nanoparticles of 1.2 nm in diameter contain ~59 zinc atoms (47 atoms in the shell and 12 atoms in core) [4]. The freshly prepared zinc nanoparticles may be all in the non-oxidized state and produce the maximal olfactory enhancement. The present XPS data showed that in one day, 3% of zinc atoms of the primarily-elemental zinc nanoparticles, or ≈2 atoms are ZnO. Being in the shell of nanoparticles, ZnO may cause an initial decline in the olfactory enhancement. After a further storage, zinc atoms diffused from the shell to the core and no longer interacted with olfactory receptors. The detrimental effect of ZnO on olfactory enhancement could be similar to that reported for cancer cells treated with the non-thermal atmospheric-pressure plasma particles that caused cell death by alteration of antioxidant activity [61]. Likewise, the polymeric membrane with ZnO particles is described to offer antibacterial activity [62]. The non-monotonic dependence on particle size, shape, support, composition, and oxidation state was found in the catalytic properties of metal nanoparticles [63] and the effects of metal nanoparticles on biological systems [64].

The mechanism of the amplified enhancement by PEG400 coating of zinc nanoparticles exposed to elevated temperatures is similar to that of the enhancement by ZnPEG400 nanoparticles subjected to long-term storage at 278 K (5 ^o^C). The XPS spectra of ZnPEG400 subjected to elevated temperature showed similar features to the Nanoparticles stored for longer times. These observations provide evidence for the large number of covalently bound species and production of a conjugated electron system of zinc nanoparticles and PEG400 molecules. The mechanism of the amplified enhancement produced by the ZnPEG400 after heating are similar to those described for these particles after prolonged storage. Although PEG reduces oxidation of elemental zinc both for 400 g/mol and 1000 g/mol molecules, the PEG1000 coating causes a considerable increase in the coating thickness which consequently reduces the enhancement of the odorant response.

We can conclude that small ~1.2 nm diameter non-oxidized primarily-elemental zinc nanoparticles produced enhancement of responses to odorants, as verified by physiological experiments, whereas air-oxidized zinc nanoparticles inhibits olfaction. When the zinc nanoparticles were covered with thin layers of polyethylene glycol of molecular weight 400 g/mol and 1000 g/mol (ZnPEG400 and ZnPEG1000), the PEGylation preserved the nanoparticles from oxidation while conserving their crystalline structure. PEGylation maintains the ability of the zinc nanoparticles to enhance the olfactory response to the odorant. The polyethylene glycol molecules by themselves produced a small olfactory response inhibition when delivered with the odorant, and did not produce a noticeable effect when applied without odorant. The ZnPEG400 nanoparticles continued to manifest the amplified enhancement of odorant response even after a 300-days storage at 278 K (5 ^o^C), while ZnPEG1000 nanoparticles produced an inhibition under the same conditions. ZnPEG400 withstood two-day storage at 303 K (30 ^o^C) and 323 K (50 ^o^C) and evoked the adequate enhancement of olfaction, but ZnPEG1000 showed a reduced enhancement at the same conditions. The PEG400 appeared to be far better for preservation of properties of zinc nanoparticles for long term storage and elevated temperatures. The enhancement and inhibition of olfaction by zinc nanoparticles preserved by PEGylation were consistent with Turin’s model of olfaction. The novelty of this work is that biocompatible and safe PEG400 coat of zinc nanoparticles provides both preservation of particles and amplification of olfactory enhancement. Future olfactory experiments with the intranasal delivery of protected zinc nanoparticles to a transient chemically acquired anosmic rodent model and a cognitively impaired Alzheimer’s rodent model will test the pre-clinical validity of smell enhancement described in this work.

## Supporting information

S1 Supporting Information The S1 Supporting Information show details of methods and calculations (PDF). (PDF)
