# Supplementary material for: PEGylation of zinc nanoparticles amplifies their ability to enhance olfactory responses to odorant
Source: PLoS One. 2017 Dec 20;12(12):e0189273. doi: 10.1371/journal.pone.0189273 (PMC5738065; doi:10.1371/journal.pone.0189273)
Supplement: S1 Supporting Information — (PDF) [file pone.0189273.s001.pdf]

PEGylation of zinc nanoparticles amplifies their ability to enhance olfactory responses to odorant

Melissa Singletary[1], Samantha Hagerty[1], Shin Muramoto[2], Yasmine Daniels[2], William A MacCrehan[2], Gheorghe Stan[2], June W. Lau[2], Oleg Pustovyy[1], Ludmila Globa[1], Edward E. Morrison[1], Iryna Sorokulova[1], and Vitaly Vodyanoy[1*]

[1] Department of Anatomy, Physiology and Pharmacology, Auburn University College of Veterinary Medicine, Auburn, Alabama, USA

[2] Material Measurement Laboratory, National Institute of Standards and Technology, Gaithersburg, Maryland, USA

*Corresponding Author: Vitaly Vodyanoy, 109 Greene Hall, Auburn University, Auburn, AL 36849, USA, e-mail: vodyavi@auburn.edu; tel.; +1-334-844-5405.

Supplementary materials

Striking Image

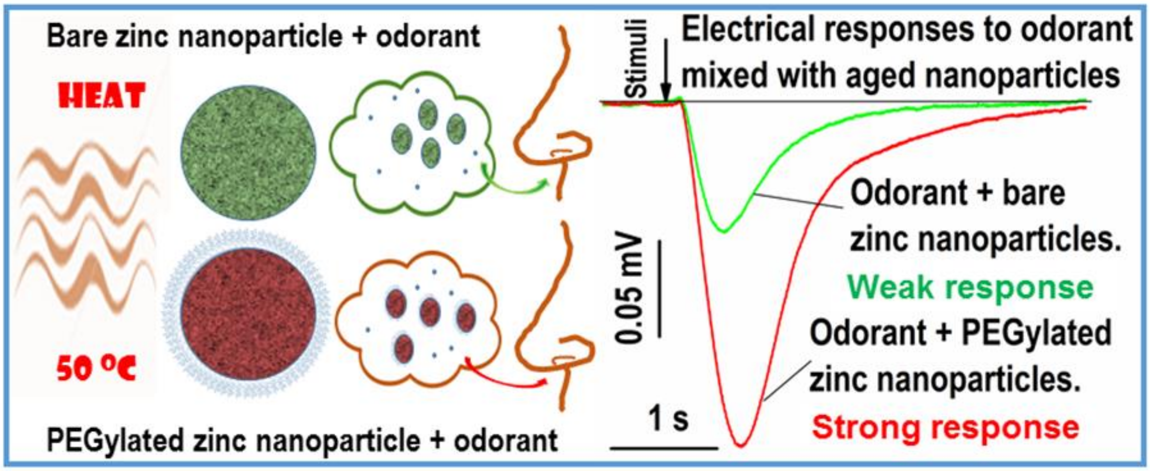

Number of monomers in poly-ethylene glycols (PEGs)

The structural molecular formula of PEG is

$H\text{-}(O\text{-}CH_2\text{-}CH_2)_n\text{-}OH$

The molecular mass of the polymer is a sum of masses of H, OH and N×(mass of the monomer) =18 + N×44. Therefore, the number of monomers in the PEG with a molecular mass of M is equal

N=(M-18)/44.

Consequently, the number of monomers in PEG400=(400-18)/44≈9 and in PEG1000=(1000-18)/44≈23.

Thickness of the PEG layer on the surface of nanoparticle

Using the area per PEG single chain of a surface of a nanoparticle [1] the estimate of the PEG layer thickness (L) can be done by the following equations[2]:

$$L = \frac{Na^{5/3}}{D^{2/3}}, \qquad (1S)$$

Where *a*=0.35 nm is a length of a single PEG monomer, D – the distance between the PEG's chains defined by the equation:

$$D = 2\sqrt{\frac{A}{\pi}} \qquad (2S)$$

Where *A*=1.28 nm^2 for the PEG400, and *A*=2.91 nm^2 for the PEG1000.

From equations (1S) and (2S) follows that L_{400}=1.32 nm and L_{1000}=2.28 nm.

The transmission probability of electron transfer through the PEG layer

The transmission probability *T* of electron with energy *E*, mass m through the potential barrier of height U_o and thickness *L* can be approximated by the equations [3]:

$$T = \frac{16E(U_o - E)}{U_o^2}\exp(-2\alpha L) \quad (3S)$$

$$\text{Where } \alpha = \sqrt{\frac{2m(U_o - E\}}{h^2}} \quad (4S)$$

h is Plank's constant.

For zinc nanoparticles of 1.2 nm in diameter, the electron energy (*E*) was estimated to be 79.6 meV [4]. The potential barrier of PEG layer (U_o) varies depending of method between 0.1 and 0.2 eV[5-7]. The transmission probability (*T*) of electron through PEG400 layer of L=1.32 nm and U_o=0.101 eV [7] is calculated by equations 3S and 4S.

$$\frac{16E(U_o-E)}{U_o^2}=\frac{16(0.0796eV)(0.101eV-0.0796eV)}{(0101\ eV)^2}=2.6718$$

$$\alpha L=(1.32 \times 10^{-9}m) \times \sqrt{\frac{2(9.11\times10^{-31}kg)(0.101eV-0.0796eV)(1.6\times10^{-19}J/eV)}{(1.055\times10^{-34}J.s)^2}}=0.99295$$

The transmission probability then for PEG400 layer

T_{400}=2.6718exp[-2(0.99295)]= 0.36673.

Similar calculations for 1.2 nm zinc nanoparticle covered with PEG1000 layer give

T_{1000}=0.08652.

The transmission probability is a strong function of the potential barrier (U_o). For example, when U_o=0.2eV[5, 6] , T400 and T1000 are 0.0345 and 0.00112, respectively.

Energy of activation of zinc nanoparticle oxidation

The Arrhenius equation [8] can be used to express the temperature dependence of the first-order activation kinetics:

$$k = Ae^{-\frac{E_a}{RT}} \qquad (5S)$$

where R is the universal gas constant, E_a represents the apparent activation energy, and A -- the pre-exponential Arrhenius factor. Taking the logarithm of Eq. (5S) yields:

$$\log k = -\frac{E_a}{2.303R} \times \frac{1}{T} + \log A \qquad (6S)$$

If the logarithm of k in Eq. (5S) is plotted against the reciprocal of temperature, 1/T, then the slope of this graph yields the activation energy (E_a), the thermal activation level of transitions from not-oxidized to oxidized atoms.

References

1. Butterworth MD, Illum L, Davis SS. Preparation of ultrafine silica- and PEG-coated magnetite particles. Colloids and Surfaces A: Physicochemical and Engineering Aspects. 2001;179(1):93-102.
2. Perry JL, Reuter KG, Kai MP, Herlihy KP, Jones SW, Luft JC, et al. PEGylated PRINT Nanoparticles: The Impact of PEG Density on Protein Binding, Macrophage Association, Biodistribution, and Pharmacokinetics. Nano letters. 2012;12(10):5304-10.
3. Griffiths DJ. Introduction to Quantum Mechanics. Upper Saddle River, New Jersey: Prentice Hall; 1995. 394 p.
4. Hagerty S, Daniels Y, Singletary M, Pustovyy O, Globa L, MacCrehan WA, et al. After oxidation, zinc nanoparticles lose their ability to enhance responses to odorants. Biometals 2016;29(6):1005-18.
5. Smolne S, Weber S, Buback M. Propagation and Termination Kinetics of Poly(Ethylene Glycol) Methyl Ether Methacrylate in Aqueous Solution. Macromolecular Chemistry and Physics. 2016;217(21):2391-401.

6. Sannaningannavar FM, Navati BS, Ayachit NH. Activation energy (ΔG*), enthalpy (ΔH*), and entropy (ΔS*) of poly(ethylene glycol) using Higasi method. Polymer Bulletin. 2016;73(6):1689-700.
7. Kabra K, Sonkamble AA, Wananje KH, Kumbharkhane AC, Sarode AV. Dynamics of polymer matrix in non-polar solvent Using TDR Technique. BIONANO FRONTIER Vol 8 (3) December 2015. 2015;8(3):378-80.
8. Segel IH. Biochemical calculations. 2d ed. ed. New-York: John Wiley & Sons; 1976.
